# Supplementary material for: ‘I wanted to go, but they said wait’: Mothers’ bargaining power and strategies in care-seeking for ill newborns in Ethiopia
Source: PLoS One. 2020 Jun 5;15(6):e0233594. doi: 10.1371/journal.pone.0233594 (PMC7274445; doi:10.1371/journal.pone.0233594)
Supplement: Attachment — Submitted filename: Response to Reviewers.docx [file pone.0233594.s002.docx]

# Response to Reviewers

Dear Jennifer Yourkavitch and PlosOne editorial team,

We thank you for the opportunity to revise and resubmit our article. The comments from the reviewers were highly relevant and helpful, and we have sought to emphasize the concerns raised by the reviewers in our revised manuscript.

In this rebuttal letter we clarify changes made and responses to the journal requirements and comments made by the reviewers. In addition to this response to reviewers we attach two files: 1) Revised manuscript *with* tracked changes, 2) Manuscript (Revised manuscript *without* tracked changes). Responses to the reviewers’ comments in this letter are marked in red.

Throughout revision, we made additional edits to improve the paper, language and coherence of the text (all changes shown in the marked version of the manuscript).

Concerning data availability, we strongly support PLOS ONE’s efforts to make data publicly available. However, in this qualitative study ethical concerns make it impossible for us to make these data available, as outlined in the following data availability statement on page 27:

**“The study is based on** qualitative data from interviews and FGDs. The data cannot be made publicly available or available upon request as this would compromise ethical concerns for participants’ privacy and confidentiality.”

We thank you again for considering our manuscript for publication. Please let us know if you have any questions or comments.

Best wishes,

The authors

Journal Requirements:

1. When submitting your revision, we need you to address these additional requirements.
Please ensure that your manuscript meets PLOS ONE's style requirements, including those for file naming. The PLOS ONE style templates can be found at
<http://www.journals.plos.org/plosone/s/file?id=wjVg/PLOSOne_formatting_sample_main_body.pdf> and <http://www.journals.plos.org/plosone/s/file?id=ba62/PLOSOne_formatting_sample_title_authors_affiliations.pdf>

PLOS ONE's style and formatting requirements have been addressed following revision of the manuscript, please see attached article.

2. Please include a copy of the interview guide used in the study, in both the original language and English, as Supporting Information, or include a citation if it has been published previously.

We have uploaded the interview guides as Supporting Information (Interview guides family members, interview guide health care workers, FGD guide community members, FGD guide health care workers).

3. We note that you have indicated that data from this study are available upon request. PLOS only allows data to be available upon request if there are legal or ethical restrictions on sharing data publicly. For information on unacceptable data access restrictions, please see <http://journals.plos.org/plosone/s/data-availability#loc-unacceptable-data-access-restrictions>.
In your revised cover letter, please address the following prompts:
a) If there are ethical or legal restrictions on sharing a de-identified data set, please explain them in detail (e.g., data contain potentially identifying or sensitive patient information) and who has imposed them (e.g., an ethics committee). Please also provide contact information for a data access committee, ethics committee, or other institutional body to which data requests may be sent.
b) If there are no restrictions, please upload the minimal anonymized data set necessary to replicate your study findings as either Supporting Information files or to a stable, public repository and provide us with the relevant URLs, DOIs, or accession numbers. Please see <http://www.bmj.com/content/340/bmj.c181.long> for guidelines on how to de-identify and prepare clinical data for publication. For a list of acceptable repositories, please see <http://journals.plos.org/plosone/s/data-availability#loc-recommended-repositories>.
We will update your Data Availability statement on your behalf to reflect the information you provide.

Please see explanation provided on page 2 in letter: Concerning data availability, we strongly support PLOS ONE’s efforts to make data publicly available. However, in this qualitative study ethical concerns make it impossible for us to make these data available, as outlined in the following data availability statement on page 27:

**“The study is based on** qualitative data from interviews and FGDs. The data cannot be made publicly available or available upon request as this would compromise ethical concerns for participants’ privacy and confidentiality.”

4. Please include a title for Table 1.

The following title has been included for the Table, now presented as Table 2: Basic characteristics of primary caretakers experiencing newborn illness and death, please see page 10.

Additional Editor Comments:

Please respond to the reviewers' comments and make appropriate revisions. Please be sure to thoroughly review and edit the manuscript for English grammatical accuracy and correct usage.

Please see responses and revisions as outlined in detail below. Specific attention has been given to English grammar and language, and the paper has gone through professional language editing.

Reviewers' comments:

Reviewer's Responses to Questions

**Comments to the Author**

1. Is the manuscript technically sound, and do the data support the conclusions?

The manuscript must describe a technically sound piece of scientific research with data that supports the conclusions. Experiments must have been conducted rigorously, with appropriate controls, replication, and sample sizes. The conclusions must be drawn appropriately based on the data presented.

Reviewer #1: Partly

Reviewer #2: Yes

2. Has the statistical analysis been performed appropriately and rigorously?

Reviewer #1: I Don't Know

Reviewer #2: N/A

3. Have the authors made all data underlying the findings in their manuscript fully available?

The [PLOS Data policy](https://eur01.safelinks.protection.outlook.com/?url=http%3A%2F%2Fwww.plosone.org%2Fstatic%2Fpolicies.action%23sharing&data=02%7C01%7C%7Ca8839a9c9f2d441fb37008d76c251659%7C1faf88fea9984c5b93c9210a11d9a5c2%7C0%7C1%7C637096778931462171&sdata=N51kwSLnc8XrzTz%2Bd8PFFzJlg%2FDk8HQ7vi2Zf8B9hxI%3D&reserved=0) requires authors to make all data underlying the findings described in their manuscript fully available without restriction, with rare exception (please refer to the Data Availability Statement in the manuscript PDF file). The data should be provided as part of the manuscript or its supporting information, or deposited to a public repository. For example, in addition to summary statistics, the data points behind means, medians and variance measures should be available. If there are restrictions on publicly sharing data—e.g. participant privacy or use of data from a third party—those must be specified.

Reviewer #1: No

Reviewer #2: No

4. Is the manuscript presented in an intelligible fashion and written in standard English?

PLOS ONE does not copyedit accepted manuscripts, so the language in submitted articles must be clear, correct, and unambiguous. Any typographical or grammatical errors should be corrected at revision, so please note any specific errors here.

Reviewer #1: No

Reviewer #2: No

5. Review Comments to the Author

Please use the space provided to explain your answers to the questions above. You may also include additional comments for the author, including concerns about dual publication, research ethics, or publication ethics. (Please upload your review as an attachment if it exceeds 20,000 characters)

**Reviewer #1:**

This paper employs a qualitative assessment of women’s roles in making decisions about and seeking care for ill newborns. The paper drew from interviews and focus group discussions from mothers and community members and conclude that women take various approaches in care seeking. The topic is of importance to policy and program stakeholders and draws attention the need to consider women’s empowerment-related barriers to seeking care for their newborn in the design of recommendations and programs.

Although there are a multitude of factors that are at play in empowerment, the paper focuses mostly on the different types and mechanisms of decision-making thereby downplaying the role of other critical factors, for example, poverty and financial constraints, especially in the results section. Of additional concern is the lack of clarity in the in the methods section. It should be noted up front, with a forthright description of the circumstances under which the interviews and focus groups were conducted, with more than just the citation of previously published articles based on the same study. Of lesser but nonetheless important note, throughout the paper, the writing can be made more succinct, precise, and should undergo a rigorous content and copy-editing.

We thank the reviewer for raising these important concerns on the structural issues that shape decision making and for pointing at how the previous version of the paper did not present the context, recruitment or data collection in detail. We respond to the specific issues in details below. In our revision, we have highlighted how structural factors influence care seeking and decision making and further put particular focus on language. The paper has also been through professional academic and language editing.

Major points:
A. The literature review is not entirely convincing of a gap in the literature or for the need of the paper.

We thank the reviewer for their comments on the introduction and the literature presented. The background and introduction section has gone through comprehensive revision (page 5-8). We hope the revised background provide a more convincing presentation of the current knowledge and existing research gaps. Still, as the paper is an empirical article and not a comprehensive literature review on mothers’ roles in decision making and strategies in care-seeking, our presentation is limited to what we find as most relevant for the study objectives. We strategically discuss findings from eleven studies of particular relevance (reference 9-19). In our revision, we have tried to present three sources of literature that provide the starting point for the article’s focus on the mother as key in health care-seeking for ill newborns: linking and questioning 1) the focus and assumptions in international and Ethiopian health policies and guidelines (sub-paragraph 1, introduction), 2) existing literature on primary care givers care seeking (sub-paragraph 2, introduction, and 3) Relevant development and empirical findings from Ethiopia (Sub-paragraph on Ethiopia).

B. Line 158 the authors say 41 in depth interviews, but it looks like there are 21 based on Table 1. Also, what is the breakdown of number of people and type of person (health workers, community members, care takers) in each FGD? Were the focus group participants the same as the in-depth interview participants? If the groups were homogenous, were there different questions for different type of people (health workers, community members, care takers)? Is there an appendix of questions asked? Over what period of time were these FGDs/interviews conducted? Some quotes were described by urban/rural locality- was there disaggregated analysis done? If so, that needs to be described in methods. Even if the study design is described elsewhere, this section needs more information and to be clarified. Perhaps of the utmost concern is that data collection was conducted for another study, the main study objectives or potential implications of this were barely mentioned.

We thank the reviewer for this important and clarifying comment. We realize that the previous version of the paper was not clear about recruitment of participants, and that further information was needed on the overall study. By updating the methods section (including 1) one new table 1 and 2) revising table 2) on page 8 to 11, we hope the text and tables give a more in-depth presentation of the data collection and recruitment and addresses the concerns raised, please see revised paragraph:

“The study was carried out through the Butajira Rural Health Program (BRHP). The BHRP is a health and demographic surveillance system which has monitored and collected data on demographics and vital events in its ten kebeles (villages) in the area since 1987.[30] Participants were recruited from the BHRP catchment area and two hospitals and affiliated health centres surrounding Butajira. The data comprised 41 in-depth interviews with primary care-takers and health workers and seven focus group discussions (FGDs) with community members (Table 1).

With emphasis on decision making processes and care-seeking, in-depth interviews (11) in hospital with primary care-takers experiencing newborn illness (admitted at hospital > one day) were followed by subsequent interviews at home (9). Through retrospective identification of cases of newborn deaths the previous year, we talked with primary care-takers about their experiences when babies fell ill. Baseline characteristics of primary care-takers are described in Table 2. To better understand how decision making processes related to care-seeking for ill newborns, we approached community members (5 FGDs) and health care workers (11 interviews and 2 FGDs) (Table 1). Identification of community members was done in collaboration with BHRP, independent from recruitment primary care-takers.”

To clarify the paper’s relation to the previous study, we revised the introductory paragraph on methods on page 8:

“A larger qualitative study on decision making and resource allocation on newborn health in Ethiopia was conducted in the autumn of 2015.[26,27] The study was carried out in Butajira, located 130 km south of the capital Addis Ababa. Skilled delivery and postnatal check-ups in the region is low (26% and 17%, respectively), but similar to national coverage.[22,28] Four out of ten women in Butajira have experienced child death.[29] In this poverty-stricken context, care-seeking for ill newborn was commonly delayed, as ill newborns were perceived to be of high risk of dying and health care seeking was costly.[26,27]”

The Supporting Information files (NEW) included the guides for the in-depth interviews and focus group discussions. We are sorry that this information was not provided in the previous version of the article.

C. The results section was not clearly organized, were not always well supported by quotes, and the interpretations presented at times misrepresented the issue. For example:

We thank you for your comments, we have revised the results section by adding additional quotes to support our findings (page 11 - 21), please see specific responses below (a-e).

a. In section on identifying problem: there is no quote at all. The subject in this paragraph seems to switch from women to a specific woman. It should more explicitly state whether these were themes or based on single woman’s experiences.

We thank the reviewer for this comment. To avoid a too long paper, we had reduced the number of quotes in the previous version of the paper. We have added the following quote text (page 12) illustrating this subtheme:

“One mother explained how she discovered that her newborn was not doing well the first days following birth:

He was breastfeeding at that time, but he suddenly stopped. He got really sick. I told them [the neighbours]. (Interview, mother experiencing newborn death, 17)”

b. In section on depending on decisions: the interpretations of the quotes presented and/or the entirety of the discussions diminish the power dynamic. What comes across in the quotes is that women do not have the authority in the relationship to decide to seek care. Yet the author uses words like “interdependence” (line 233) and “the husbands role made him responsible” (line 235) and “decisions where and how to seek care were not necessarily theirs to make” really undermine and downplay the disempowerment issues that are elucidated here. It’s a power dynamic that, based on the quotes presented, should be highlighted.

We thank the reviewer for this feedback. We agree with the reviewer’s interpretation that power and empowerment are key in understanding women’s decision making and care seeking strategies. We have revised the sentences on page 13, but would like to keep the interpretation and discussion power dynamics to the discussion section (page 21 - 22). In this way, we make it clearer for the reader what the participants said, and how we interpret these findings.

Page 13, line 249:

“FGD participants described the importance of the husband’s decision making role and power.”

c. Line 258- add in older relatives or neighbors “thought they” knew whether to seek… It appears they didn’t actually know if the baby was actually sick and were advising not to go because they misunderstood medicine.

Thank you for your suggestions, the sentence has been rewritten and reads Based on earlier experience, older relatives or neighbours often thought they knew whether and where to seek treatment” (page 14, line 273).

d. Quote starting on line 323 isn’t relative to taking care of a sick baby, it’s about not getting to the facility in time for delivery, and what sounds like a quick labor. I wouldn’t include the quote here, it’s not relevant to the focus of the paper.

We thank the reviewer for this useful feedback. We agree that the quote illustrates several of the challenges the woman faced (including rapid progression of labour, birth during the night without access to transportation, low bargaining power). We have taken the quote out, and provide a different quote from our analysis, please see page 17:

“One mother realized that her baby was ill, but was all alone and did not get support from anyone following birth. She accepted that she could not go to the hospital even after recognizing that the baby was not well:

It was only me who wanted to go. No one said “go to the hospital”. She [her mother] didn’t tell me to go - because the baby was getting tired and not taking the breast. I thought if she would glucose she would get better. But no one said anything, I was all alone. (Interview, mother experiencing newborn death, rural, 9)”

e. Maneuvering and Action and opposition sections: it’s unclear in these two sections what applies to which place of residence. Importantly, the mention in these two sections begs the question about urban/rural differences in other sections.

The reviewer rightly points at the potential differences between rural and urban areas. We have revised the results section with special attention to rural/urban differences, including providing information by each quote used on rural/urban location, and additional information in Table 1 and 2. As pointed out in the revised Results and Discussion sections, these strategies were only available to some women --- often urban, more educated and with access to finances. As this is a key finding of the study, we thank the reviewer for helping us to highlight this issue. As the aim of the study was not to examine rural and urban differences, we cannot make a final conclusion on how urban/rural residence matter, but we argue that it is one of the factors that influence mother’s bargaining power and room of action.

D. The discussion could be more succinct and organized, with additional emphasis on the confluence of factors that relate to women’s status, empowerment, and ability to seek care, including poverty, education, urban/rural location, and access to care. This is briefly mentioned lines 497-505 but that seems to be the crux of the study.

We thank the reviewer for this crucial comment, which links to comment C. We have revised the Discussion with emphasis on highlighting the structural issues that shape and underpin mother’s bargaining power and decision making roles, please see changes page 21-23.

Minor points:
1. Introduction: Although I am not completely familiar with the literature on the topic, I would assume this topic-women’s empowerment as it relates to care-seeking has been well-studied. The article mentions three distinct studies but spends only one sentence on tying them together. I question if this is a comprehensive review, and if more summary on the topic including other relevant literature can be added.

We thank the reviewer for this comment. We have revised the introduction, as pointed out on page 5-6. While women’s empowerment and agency is a crucial topic, including a full review of the existing literature goes beyond the scope of this specific study with focus on decision making with regards to the newborn.

2. Suggest moving the Study Context section from Methods to Intro

We thank the reviewer for the suggestion. In our revision of the manuscript, we have moved the section providing contextual background about Ethiopia to the introduction. However, we lean towards keeping the description of Butajira and specific study context in the methods section.

3. What does treating illness through community mobilization mean ln 135-136?I see this as deployment of CHWs, not necessarily community members that are discussed in this paper, so it seems like this section may be well suited in the recommendation as a way to combat the way the culture interferes with seeking care.

We thank the reviewer for this comment. In Ethiopia, the health development army (community members) and health extensions worker are both parts of the strategy to improve newborn health, as clarified in the revised draft on page 7 (line 127-129):

“Community-based newborn care aims to identify and treat illness through community mobilization, and it is delivered through the Health Extension Programme (health programme) and the Women’s Development Army (community-based programme).”

In this way, communities are encouraged to seek care when identifying illness. While we agree with the reviewer that the role of community member is crucial, we argue that information is not enough to directly impact women’s financial bargaining power. However, access to information – and knowledge as a source of bargaining power or shared bargaining power among groups of women within the community – may influence what care seeking options that are seen as available. While this is of interest, we did not study the women’s development army nor did any participants mention it, and therefore we are hesitant about making specific policy recommendations.

4. In Table 1, the stars don’t really align with the words that they’re placed next to. It’s confusing to read like this, maybe they should be placed next to the respective column heading, and then the total number of primary care takers can be a line at the bottom of the table.

We thank the reviewer for raising this issue. We have revised the use of stars and text in the table, and hope that it is easier to read now, please see Table 2, page 10.

5. There are many instances in which the choice of words was questionable, which may either be a language/translation issue or stylistic, for example, using the word stakeholder to reflect care takers or community members (stakeholder more commonly refers to individuals or agencies invested in a program or policy). In other instances, there were issues with subject-predicate agreement, for example, line 274 (decisions to seek care aren’t costly, but medical care can be). Regardless, the paper should undergo more rigorous content and copy-editing.

We thank the reviewer for his/attention to the language and choice of words in the paper. We have taken out the word stakeholder in the article. The paper has gone through revision with focus on improving the language and professional language editing.

6. FGD spelled out in some places, acronym used in others
Thanks for your comment, we have revised the text and use FGD after the introduction in the abstract and introduction.

7. In the discussion, lines 425-459 is one big paragraph that should be broken out by key messages the authors are trying to convey that highlight the main findings of the study.

Thank you for this suggestion, the key messages are presented in three new paragraphs in the revised version of the article. We hope these changes make the messages and discussion more easy to follow, please see page 21-23.

**Reviewer #2:**

The manuscripts makes an important contribution to the literature on the participation of mothers in the care-seeking process of ill children. However there are several aspects of the manuscript that require improvement, revision and clarification.

Introduction

-Overall the writing quality can be improved - there is need for further editorial and grammatical corrections.

We thank you for this comment. Throughout the manuscript we have done careful revision of language and grammar and the paper has been read by a native speaker.

-There is need for consistency in terminology or definitions of unclear terminology. For example, line 74 states that there is emphasis on the mother to 'improve newborn health', yet the prior discussion was on care-seeking, with the aim to improve care-seeking behavior.

Thank you for your comment. We agree that the previous sentence may have been misleading. A comprehensivly revised introductory paragraph now reads, line 70-72:

“…the responsibility placed on the mother as the key to improving newborn health and care-seeking for ill babies may not be supported by her capability to fill this function.”

-Line 77-78- define what is meant by health behaviour

Thank you for this clarifying comment. Through revision of the manuscript, the sentence has been taken out.

-Line 111-114, are there any citations to support this statement

We thank the reviewer for this comment. The introduction has been revised, but the claims are being presented on page 6:

“While studies emphasize the importance of household and family-level factors and early recognition of illness for ill newborns,[17-19] little is known about the roles and strategies taken by the mother in real-life decision making. We argue that this requires specific attention, as it is often assumed—explicitly or implicitly—that the mother as the primary care-taker will take action once she has identified a danger sign of newborn illness.[7,17]”

-Line 126-127, 71% seems high given the context of the rest of the discussion, is it possible to provide additional context based on this reference

While this number seems high, the DHS state that they were “actively involved in household decision making on health care, household purchases and visits”, but do not describe how they were involved. Our study illustrate that they were involved in identifying illness, but were not considered the main decision makers. In this way, our study nuance how yes/now questions may not be sufficient to understand how decisions are made.

Study design

The reviewer has pointed at several important concerns about the study design of our descriptive qualitative study. The method section has been revised and should provide information about data collection process, recruitment and analysis (see detailed responses below).

-who were the informants and how were they identified

Please see revised methods section (page 8-11), and details provided in Table 1(NEW!) and 2 (updated).

-what was the decision-making process of following some of the primary care takers home and not others, how was the determination made on who would be followed vs not

We aimed to follow all cases of newborn illness identified in the hospital, but was only able to that in 9 out of 11 cases due to logistical challenges (more than a day of travelling).

-how were FGD participants selected

FGD participants were selected with focus on background and location, see table 1 (NEW):

**Focus group discussions (FGDs; 4-8 participants per group)**

- *Health workers*
  - Nurses and midwifes (urban, mix of male and female)
  - Health extension workers (rural and urban, all female)

- *Community members*

- Women in reproductive age with child <1 year (urban, all female)
- Women in reproductive age with child <1 year (rural, all female)
- Husband with wife in reproductive age with child <1 year (rural, all male)
- Grandmothers (rural, all female)
- Religious leaders and elders (urban, all male)

-what was the composition of FGD participants, at minimum- male vs. female breakdown

Please see last response and Table 1.

-provide more description on the thematic analysis, how was it conducted ?

We have updated the description our analysis on page 10-11:

“Data were analysed drawing upon thematic analysis.[31] Preliminary topics were identified throughout fieldwork and were then discussed in following interviews, FGDs and in observation. The transcribed and translated data material was coded and categorized (NVivo 11 software) to identify core themes and patterns. Initial codes, categories, themes and patterns were discussed within the team. An inductive-deductive approach was taken, going back and forth between transcriptions and the coded data material with focus on manifest and latent meanings. Interpretation and analysis built upon earlier findings, describing how cultural and structural factors delayed care-seeking and impacted intra-household resource allocation.[26,27]”

-was there a documentation process of the discussions that following interviews

Daily field notes was collected during data collection.

-was a codebook developed? what was the process of developing the codebook?

We did not use a codebook in our analysis. Initial coding was done by one author (the first author). Coding and themes were discussed within the team.

-what was the coding process? were there multiple coders? if so, what was the process of determining intercoder reliability ?

Please see previous response. The first author coded the material. The team discussed the initial raw material (the first interviews), codes, themes and patterns (page 11).

-need more clarify on line 184- 'findings influenced by earlier interpretations'

We have updated the explanation, please see page 11:

“Interpretation and analysis built upon earlier findings, describing how cultural and structural factors delayed care-seeking and impacted intra-household resource allocation.[26,27]”

-there is need for more description on the consent and ethical review process, what type of consent did participants decide

We thank the reviewer for raising this important ethical concern, and have added information on page 11:

“Potential participants were informed verbally about the study, the opportunity to participate, that participation was voluntary, and that no personal data would be revealed. Informed consent (written or by fingerprint) was obtained from potential participants before commencing any interviews or FGDs.”

Results
-line 244 - who tended to be the breadwinner- outside of the husband

The sentence in the previous versions of the article was unclear, it now reads (page 14):

“Health workers explained that it was the breadwinner – most often the husband – who ultimately decided whether or not to bring the infant to the hospital and which care that was preferred: “It's the husband's role because he is the income generator of the house.”

-line 249 - most often men decide whether to go to the hospital?

Thank you for raising this clarifying question, please see revision Page 14, line 264:

“Most often it is the men decide whether to go home or to stay [at the hospital when the baby is ill].”

-line 297-298- any more explanation of how the strategies were identifies- was there any differences strategies based on respondent type

The strategies were identified through the analysis (see methods). In the revised version of the manuscript, we have included information about rural/urban location of the participant, in all quotes and and the discussion (page 23) provide more information about the strategies used (and by whom).

General-
-was there is an effort to distinguish between women that experienced a newborn death vs. those that did not- it is not clear if there are differences in their experiences of these women in terms of care-seeking behaviors?

We were not able to identify clear differences in their experiences. As outlined in the updated Table 2, 4 of 11 women facing newborn illness did not give birth in health facilities, and 5 of 10 women facing newborn illness did not give birth in facilities. While this is a highly relevant question, it was not the main focus of this study and we cannot conclude on whether their experiences differed.

- any differences in views by FGD participants ?

We were not able to differentiate clear views between different FGD participants. While this could have been of interest, it sadly went beyond the aim of the study. We welcome further research in this areas relevant for our understanding of the different strategies used.

6. PLOS authors have the option to publish the peer review history of their article ([what does this mean?](https://eur01.safelinks.protection.outlook.com/?url=https%3A%2F%2Fjournals.plos.org%2Fplosone%2Fs%2Feditorial-and-peer-review-process%23loc-peer-review-history&data=02%7C01%7C%7Ca8839a9c9f2d441fb37008d76c251659%7C1faf88fea9984c5b93c9210a11d9a5c2%7C0%7C1%7C637096778931467159&sdata=2Qgh2B1SN8hP%2Fzc5bRZYSGkkzf0KjzJ9QMsFx0z%2FUN8%3D&reserved=0)). If published, this will include your full peer review and any attached files.


If you choose “no”, your identity will remain anonymous but your review may still be made public.


**Do you want your identity to be public for this peer review?** For information about this choice, including consent withdrawal, please see our [Privacy Policy](https://eur01.safelinks.protection.outlook.com/?url=https%3A%2F%2Fwww.plos.org%2Fprivacy-policy&data=02%7C01%7C%7Ca8839a9c9f2d441fb37008d76c251659%7C1faf88fea9984c5b93c9210a11d9a5c2%7C0%7C1%7C637096778931472152&sdata=PvL4oDL7YTfXHtm6KL7iVurmGSs0MiosqYLgp9QwkCQ%3D&reserved=0).

Reviewer #1: No

Reviewer #2: Yes: Lwendo Moonzwe Davis


[NOTE: If reviewer comments were submitted as an attachment file, they will be attached to this email and accessible via the submission site. Please log into your account, locate the manuscript record, and check for the action link "View Attachments". If this link does not appear, there are no attachment files to be viewed.]
