# Supplementary material for: ‘I wanted to go, but they said wait’: Mothers’ bargaining power and strategies in care-seeking for ill newborns in Ethiopia
Source: PLoS One. 2020 Jun 5;15(6):e0233594. doi: 10.1371/journal.pone.0233594 (PMC7274445; doi:10.1371/journal.pone.0233594)
Supplement: Attachment — Submitted filename: Response to Reviewers 1.docx [file pone.0233594.s003.docx]

# Response to Reviewers

Dear Jennifer Yourkavitch and PlosOne editorial team,

We thank you for the opportunity to resubmit our article. We have sought to emphasize the concerns raised by the reviewer and the editiorial team in our revised manuscript, with focus on the methodological aspects.

In this rebuttal letter we clarify changes made and responses to the journal requirements and comments made by the reviewers. In addition to this response to reviewers we attach two files: 1) Revised manuscript *with* tracked changes, 2) Manuscript (Revised manuscript *without* tracked changes). Responses to the reviewers’ comments in this letter are marked in red.

Following revision, we made some additional change to improve the paper, language, coherence of the text and modified Table 1 (all changes shown in the marked version of the manuscript).

There are no updates on our financial disclosures. While we support laboratory protocols, these are not applicable for a qualitative study such as ours.

We thank you again for considering our manuscript for publication. Please let us know if you have any questions or comments.

Best wishes,

The authors

Additional Editor Comments (if provided):

Thank you for this revision, which addressed many of the reviewers' comments. There are still serious concerns about the analysis, which does not conform to qualitative research standards. At a minimum, please invite a second person to code and analyze the data so that there is some demonstrable validity to the results and conclusions. Please also create and use a code book. And then describe fully the methods you used to re-analyze the data. Describe the themes that emerged from the analysis. Please address and respond to all of the reviewer's comments.
Qualitative data can be made publicly available without identifying information. You can provide tables or matrices with raw information, organized by themes.

The reviewer raised several important issues concerning our presentation of the thematic analysis, the process of analysis and its validity. The revised version of the manuscript include a new paragraph of the methods section and new Figure (Figure 1) that we hope respond to the requests for a clarification. As depicted in the revised manuscript the analysis involved all researchers. While one researcher led the initial coding (one step of a six phases process), the others authors (led by the last author) contributed substantively in discussions of codes, sub-themes and themes. Figure 1 further outlines the themes and subthemes of this study (phase 5). Though we referred to thematic analysis in the previous version of the manuscript, we understand that the last version of the manuscript did not provide details on the rigorousness of the analysis and approach taken. As Braun and Clark writes, the thematic map (phase 5, Figure 1) is not as detailed as a codebook, but provide the reader with an understanding of the themes and subthemes.

We have also modified Table 1 to a more reader-friendly table.

We hope these changes have addressed the concerns raised by the editor and reviewer on the validity of the study. Please see detailed response below and in the revised version of the manuscript.

[Note: HTML markup is below. Please do not edit.]

Reviewers' comments:

Reviewer's Responses to Questions

**Comments to the Author**

1. If the authors have adequately addressed your comments raised in a previous round of review and you feel that this manuscript is now acceptable for publication, you may indicate that here to bypass the “Comments to the Author” section, enter your conflict of interest statement in the “Confidential to Editor” section, and submit your "Accept" recommendation.

Reviewer #2: (No Response)

2. Is the manuscript technically sound, and do the data support the conclusions?

The manuscript must describe a technically sound piece of scientific research with data that supports the conclusions. Experiments must have been conducted rigorously, with appropriate controls, replication, and sample sizes. The conclusions must be drawn appropriately based on the data presented.

Reviewer #2: Partly

3. Has the statistical analysis been performed appropriately and rigorously?

Reviewer #2: N/A

4. Have the authors made all data underlying the findings in their manuscript fully available?

The [PLOS Data policy](https://eur01.safelinks.protection.outlook.com/?url=http%3A%2F%2Fwww.plosone.org%2Fstatic%2Fpolicies.action%23sharing&data=02%7C01%7C%7C3ab298e847344b37152608d7c4f513a4%7C1faf88fea9984c5b93c9210a11d9a5c2%7C0%7C0%7C637194429210182078&sdata=2hzXWSmrW5Xxji%2FTxli1iHyFrJR7PXWErK0kBCUCnWQ%3D&reserved=0) requires authors to make all data underlying the findings described in their manuscript fully available without restriction, with rare exception (please refer to the Data Availability Statement in the manuscript PDF file). The data should be provided as part of the manuscript or its supporting information, or deposited to a public repository. For example, in addition to summary statistics, the data points behind means, medians and variance measures should be available. If there are restrictions on publicly sharing data—e.g. participant privacy or use of data from a third party—those must be specified.

Reviewer #2: No

5. Is the manuscript presented in an intelligible fashion and written in standard English?

PLOS ONE does not copyedit accepted manuscripts, so the language in submitted articles must be clear, correct, and unambiguous. Any typographical or grammatical errors should be corrected at revision, so please note any specific errors here.

Reviewer #2: No

6. Review Comments to the Author

Please use the space provided to explain your answers to the questions above. You may also include additional comments for the author, including concerns about dual publication, research ethics, or publication ethics. (Please upload your review as an attachment if it exceeds 20,000 characters)

Reviewer #2: The authors have made significant improvements to the manuscript, there are noteworthy improvements to the introduction, discussion and conclusion. However, the primary concern with the manuscript upon further review is the lack of methodological and analytical rigor, which is not of the standard required for publication. The authors note that a codebook was not developed and the coding done by one author with discussion by team. There is limited discussion on the process used for coding and analysis and thus it is not possible to assess the validity of the process.

Specifically the reliability and validity of the research findings are called into question without the details of the analytical process. For example, the authors discuss conducting thematic analysis however it is not clear which themes emerged, are the only themes that emerged those that are discussed in the paper? Were there other themes that were not included, if so what was the process for deciding what would be included? Since coding was conducted by a single author, would the same themes be evident to others? Was there an attempt to create objectivity in the analysis? Where any of the analytical tools that are available in Nvivo utilized for the analysis? Were interviews recorded and transcribed? Or were transcripts based on notes? What was the quality of these transcripts? Where there any data quality related challenges in the transcription and translation process? If so how were these mitigated?

We thank the reviewer for raising concerns about the analytical process as well as the reliability and validity of the findings. In our revision of the methods section, we have aimed to address the important issues raised by the reviewer, please see new and edited sections:

Page 9-10:

“Interviews and FGDs were audio-recorded (all FGDs, 38 interviews) or notes were taken (three interviews). Transcripts were translated from Amharic to English. MM conducted quality checks in translation. Data were analysed using thematic analysis.[31] Initial analysis started during data collection with daily reviews of topics covered in interviews and FGDs. Interview and topic guides were revised to integrate new insights. The transcribed data material was analysed and coded (NVivo 11 software) to identify core themes and patterns (Figure 1). Overarching themes and sub-themes were discussed within the team after data collection and initial coding of the material. Following Braun and Clark [31, page 87], the analysis involved six phases: I: Getting familiar with the data (all authors). II: Initial coding (KHO, supported by IM). III: Searching for themes (all authors). IV: Reviewing themes (KHO, supported by IM and other authors). V: Defining and naming themes (KHO, IM, KMM) (Figure 1). VI: Producing the report (KHO, supported by all authors). An inductive-deductive approach was taken in the analysis, going back and forth between transcriptions and the coded data material in phases III-VI [31]. After the descriptive phase of the analysis, where we focused on the manifest or descriptive meanings of the data, we moved on to the interpretative phase where we searched for latent meanings of the data material [31]. For instance, a mother’s description of how “[the neighbours] insisted that he [the baby] should stay at home” was coded under the sub-theme “depending on guidance and support from neighbours and family members” under the theme “depending on others” (Figure 1). Going beyond manifest descriptions we took an interpretive approach in examining latent features and meanings of mothers’ care-seeking decisions. We understood mothers’ practices, as when avoiding advice from the elderly, as a manoeuvring strategy in navigating care-seeking [31, page 84]. Uncertainty in analysis and interpretation of the data was discussed within the team. Building on the data material and interpretation, final themes and sub-themes were developed (Figure 1).”

Page 7-8:

“Triangulation in sources of participants (primary care-takers, health care workers, community members) and data collection methods (interviews, FGDs) and member checking during data collection were conducted to enhance the study’s trustworthiness and credibility.”

We have also added a Figure (Figure 1) which details the themes and sub-themes (page xx).

The reviewer also asks whether analytical NVIVO tools were used. We have used the coding and node-functions in NVIVO, but not the advanced tools. Two of the authors (IM and KHO) were responsible for the coding and the themes and sub-themes were discussed within the team in meetings through the six phases listed in the revised methods section. While NVIVO enables several helpful features, we found in-depth discussion of the material and interpretation to be most useful in searching for latent and manifest meanings.

Although the authors have not adequately described there analytical process, they have described noteworthy findings and conclusions. Yet without this foundational aspect, the one cannot sufficiently assess the validity of these findings and conclusions.

7. PLOS authors have the option to publish the peer review history of their article ([what does this mean?](https://eur01.safelinks.protection.outlook.com/?url=https%3A%2F%2Fjournals.plos.org%2Fplosone%2Fs%2Feditorial-and-peer-review-process%23loc-peer-review-history&data=02%7C01%7C%7C3ab298e847344b37152608d7c4f513a4%7C1faf88fea9984c5b93c9210a11d9a5c2%7C0%7C0%7C637194429210182078&sdata=uyPbfg9vGTM5kfa2CB3tayXWOzSB3v5ctD7fXfJbdns%3D&reserved=0)). If published, this will include your full peer review and any attached files.


If you choose “no”, your identity will remain anonymous but your review may still be made public.


**Do you want your identity to be public for this peer review?** For information about this choice, including consent withdrawal, please see our [Privacy Policy](https://eur01.safelinks.protection.outlook.com/?url=https%3A%2F%2Fwww.plos.org%2Fprivacy-policy&data=02%7C01%7C%7C3ab298e847344b37152608d7c4f513a4%7C1faf88fea9984c5b93c9210a11d9a5c2%7C0%7C0%7C637194429210192072&sdata=5uLW%2BLyqH85gEcOwrp2tO2Q3JMq8q%2Fx11pimGdbFjbE%3D&reserved=0).

Reviewer #2: No


[NOTE: If reviewer comments were submitted as an attachment file, they will be attached to this email and accessible via the submission site. Please log into your account, locate the manuscript record, and check for the action link "View Attachments". If this link does not appear, there are no attachment files to be viewed.]


While revising your submission, please upload your figure files to the Preflight Analysis and Conversion Engine (PACE) digital diagnostic tool, [https://pacev2.apexcovantage.com/](https://eur01.safelinks.protection.outlook.com/?url=https%3A%2F%2Fpacev2.apexcovantage.com%2F&data=02%7C01%7C%7C3ab298e847344b37152608d7c4f513a4%7C1faf88fea9984c5b93c9210a11d9a5c2%7C0%7C0%7C637194429210192072&sdata=dl6vdzO%2F%2FN1S02XaRuixvL%2FeQNvi8%2FVEU3VtCsQFFEs%3D&reserved=0). PACE helps ensure that figures meet PLOS requirements. To use PACE, you must first register as a user. Registration is free. Then, login and navigate to the UPLOAD tab, where you will find detailed instructions on how to use the tool. If you encounter any issues or have any questions when using PACE, please email us at [figures@plos.org](mailto:figures@plos.org). Please note that Supporting Information files do not need this step.
