# Supplementary material for: ‘I wanted to go, but they said wait’: Mothers’ bargaining power and strategies in care-seeking for ill newborns in Ethiopia
Source: PLoS One. 2020 Jun 5;15(6):e0233594. doi: 10.1371/journal.pone.0233594 (PMC7274445; doi:10.1371/journal.pone.0233594)
Supplement: Attachment — Submitted filename: Response-to-reviewers.docx [file pone.0233594.s004.docx]

Response to editor comments

Additional Editor Comments (if provided):

Thank you for providing more information about the analysis. Since you did not reanalyze

the data with a second coder as suggested ("supported by" does not

indicate two independent coders), please include a Limitations section (perhaps

renaming and expanding the current "Methodological Considerations" section) that

acknowledges a potential lack of objectivity in the analysis due to reliance upon a

single coder. You could also state whether you believe that input from co-authors

during other steps of the analysis enabled objective discussions of the results.

Please do not indicate who did what in the Methods section of the text; remove the

initials of individuals. It is appropriate, however, to indicate where all authors

participated in a step, and also that the coding was done primarily by one person.

We appreciate your suggestion to revise the ‘Methodological considerations’ section

to acknowledge the limitations of the analytical approach taken. We have added the

following clarifying paragraph under the (new) renamed section ‘Methodological

concerns’ on page 24:

The data was analysed using thematic analysis. While it is a limitation of the

study that one author led the initial coding, a different author supported in

evaluation and re-evaluation of the codes. All authors were were involved in

the analysis, interpretation and development of sub-themes and themes

(phases III-VI), which is likely to have enhanced the trustworthiness of the

study.

Following standard assessments of validity, quality and robustness in qualiative

research1, we prefer using the terminology trustworthiness rather than objectivity

with regards to the evaluation of the analysis and its weaknesses.

In the revision of the paper, the initials of the authors have been removed, including

the following changes:

Page 9:

One of the authors conducted quality checks in translation.

Page 10:

1 E.g. see Malterud, K. (2001). "Qualitative research: standards, challenges, and guidelines." Lancet

358(9280): 483-488 and Leung, L. (2015). "Validity, reliability, and generalizability in qualitative

research." J Family Med Prim Care 4(3): 324-327.

… the analysis involved six phases: I: Getting familiar with the data (all

authors). II: Initial coding (led by one author, supported by other author). III:

Searching for themes (all authors). IV: Reviewing themes (three authors). V:

Defining and naming themes (three authors) (Figure 1). VI: Producing the

report (all authors).

Response to questions concerning the data availability statement

Dear Dr. Onarheim,

I hope this message finds you well. I note that you have recently received your

'Revise' decision on your submission, "‘I wanted to go, but they said wait’: Mothers’

bargaining power and strategies in care-seeking for ill newborns in Ethiopia" (PONED-

19-20757R2).

We further note that your Data Availability Statement reads: "The study is based on

qualitative data from interviews and FGDs. The interview transcripts cannot be made

publicly available as this would compromise ethical concerns for participants’ privacy

and confidentiality."

Could you please clarify the following as you complete the revisions to your

manuscript:

1) Would it be possible to anonymise your underlying data and make it available

either as a Supporting Information file or via an accpted data repository? We note

you previously indicated this would not be possible due to the sensitive nature of the

data but we have recently heard from Academic Editor that you may be able to

provide some of the qualitative data without revealing any identifying information.

They specifically noted the possibility of providing tables or matrices with raw

information, organized by themes.

2) If this is not possible, would you be able to provide a contact point for your

institution, IRB or data access committee, to which data access requests may be

made. In this case, we would not publish your data alongside your paper but have

the option for interested researchers to reach out to a non-author contact to acquire

data for research and replication purposes.

If either of the above options are possible, please let me know and submit you

revision accordingly. We look forward to receiving your response. Please feel free to

contact me if you have any questions or concerns.

Kind regards,

Shayan Khan

Publications Assistant, PLOS ONE | he/him

Thank you for raising the important issue concerning data availability and sharing of

data. We respond to your requests, but would like to stress that we are truly

supportive of open research and sharing of data as a mechanism to promote better

and accessible research. However, this is more difficult for qualitative data) and in

particular for our project dealing with the sensitive topic of newborn deaths and

illness. In the interest of the participants and concerns for confidentiality, we cannot

make the data from this study available.

1) We cannot make the data available through a Supporting Information file or

via an accepted data repository. The data include identifable details about the

family, the illness and deaths of newborns and decisions made by mothers

when seeking care. Some mother sought care without telling their families,

and shared their stories with knowledge that these data would not be shared.

We repeatedly stressed in our interviews that noone apart from the team of

reserachers would have access to the data and this is also expressed in the

Informed consent forms:

What will happen to the research about you?

After the interviews the material will be analyzed by the researchers. The

material will be processed without your name, ID number or other any

information that makes it possible to recognise you. Any information about

you will be kept confidential and will be anonymous in the reporting of the

results. It will not be possible to identify you when we present the results of

the study.

If we make these data available online, this would represent a breach of

confidentiality, when information provided in confidence would be disclosed to

a third party without consent.

2) Following our previous answer, sharing our data material – including parts of our data

material - would represent a breach of confidentiality. This would go against

standard research ethics standards as well as guidance from ethical committees

approving this project. In this way, we cannot meet your request to provide a focal

point for interested researchers to reach out to get access to data for research and

replication purposes. However, we would thank you for your insistence on promoting

open access to data. In our future research – on less sensitive topics – we will strive

to seek permission to share the data when possible.
